# Improvement of Clinical Outcomes of Total Aortic Arch Replacement and Frozen Elephant Trunk Surgery With Aortic Balloon Occlusion

**DOI:** 10.3389/fcvm.2021.691615

**Published:** 2021-07-26

**Authors:** Luchen Wang, Zeyi Cheng, Yunfeng Li, Junpeng Li, Hongwei Guo, Shenghua Liang, Xiaogang Sun

**Affiliations:** ^1^Aortic and Vascular Surgery Center, Fuwai Hospital, National Center for Cardiovascular Diseases, Chinese Academy of Medical Sciences and Peking Union Medical College, Beijing, China; ^2^Department of Cardiovascular Surgery, West China Hospital, Sichuan University, Chengdu, China; ^3^Qilu Hospital, Shandong University, Jinan, China; ^4^Huamei Hospital, University of Chinese Academy of Sciences, Ningbo, China

**Keywords:** aortic dissection, total aortic arch replacement, frozen elephant trunk, hypothermic circulatory arrest, aortic balloon occlusion technique

## Abstract

**Background:** Total aortic arch replacement (TAR) with frozen elephant trunk (FET) surgery provides improved long-term results, but the surgery itself is associated with higher risks compared with isolated proximal reconstructions. We applied an aortic balloon occlusion (ABO) technique to reduce the circulatory arrest (CA) time and improve other clinical outcomes.

**Methods:** All patients who underwent TAR with FET surgery (130 with ABO technique, 230 with the conventional approach) in Fuwai Hospital from August 2017 to February 2019 were reviewed in this retrospective observational cohort study. Intra- and early-postoperative results and clinical characteristics were analyzed.

**Results:** After 1:1 propensity score matching (130 cases in each group), the 30-day mortality of the ABO group and the conventional group were 4.6% and 10.8% (*p* = 0.063), respectively. Although the reduction in complications was not statistically significant, the complication rate in the ABO group was relatively low, having fewer cases of postoperative renal (23.1 vs. 38.5%, *p* = 0.007) and hepatic (12.3 vs. 30.0%, *p* < 0.001) injury, lower postoperative wake-up time (15.2 ± 23.6 h vs. 20.1 ± 26.5 h, respectively, *p* < 0.001), reduced chest tube output (176.03 ± 143.73 ml vs. 213.29 ± 130.12 ml, respectively, *p* = 0.003), lower red blood cell transfusion volume (4.98 ± 6.53 u vs. 7.28 ± 10.41 u, respectively, *p* = 0.008), and no fatal events.

**Conclusions:** The ABO technique is a simple method that can reduce the CA time and improve the recovery stage following TAR with FET surgery. The technique represents a practical strategy to treat patients with high operative risks due to its lower complication rate compared with the conventional approach.

## Introduction

Total aortic arch replacement (TAR) with frozen elephant trunk (FET) surgery is considered the most reliable method in treating type A aortic dissection (AD) in our hospital (1–3). Currently, we routinely perform TAR with FET with a lower average circulatory arrest (CA) time (17 min) with a target temperature of 26°C in non-complicated cases; this has improved postoperative results compared with the past decades. This new technique used in TAR with FET surgery can reduce the lower body CA time to 5 min on average (4). The target lowest temperature for CA has now been raised to 28°C. This study was aimed to investigate the merits of our new technique by comparing the clinical endpoints according to the International Aortic Arch Surgery Study Group (IAASSG) (5). The laboratory test results were examined to further discuss the advantages of this new technique.

## Methods

### Study Design and Patient Population

The study was approved by the institutional ethics committee of Fuwai Hospital, National Center for Cardiovascular Diseases, Chinese Academy of Medical Sciences, and Peking Union Medical College (Approval No. 2018-1069), and individual consent for this retrospective analysis was waived. The study was conducted in accordance with the Declaration of Helsinki (as revised in 2013). All data sets were represented by routine parameters from our institution that did not constitute an additional burden to the patients. The study included consecutive patients who underwent TAR with FET surgery in Fuwai Hospital from August 2017 to February 2019. A total of 130 patients underwent TAR with FET surgery via the balloon occlusion technique, while 230 patients received regular TAR with FET procedure.

### Surgical Technique of ABO Technique

A four-branched graft (Terumo; Vascutek Limited, Renfrewshire, UK) was trimmed, and the 40M All AD aortic balloon (Coda Balloon Catheter; Cook Incorporated, Bloomington, IN, USA) was inserted into an 18F Gore sheath (W.L. Gore & Associates, Inc., Flagstaff, AZ, USA), which had already been inserted into the graft. Arterial cannulation was performed through the right axillary and femoral arteries with a bifurcated arterial line from one central perfusion. Cardiopulmonary bypass (CPB) was stopped at 28°C which was the target nasopharyngeal temperature. After clamping the proximal innominate artery, the anterograde selective cerebral perfusion (ASCP) was obtained at a rate of 5–10 ml/kg/min through the right axillary artery cannula. After the Cronus stent elephant trunk (diameter: 26 or 28 mm; length: 100 or 120 mm; Cronus, MicroPort Endovascular Shanghai Co., Ltd., Shanghai, China) was released in the true lumen of the descending thoracic aorta (DTA).

**Figure 1:**
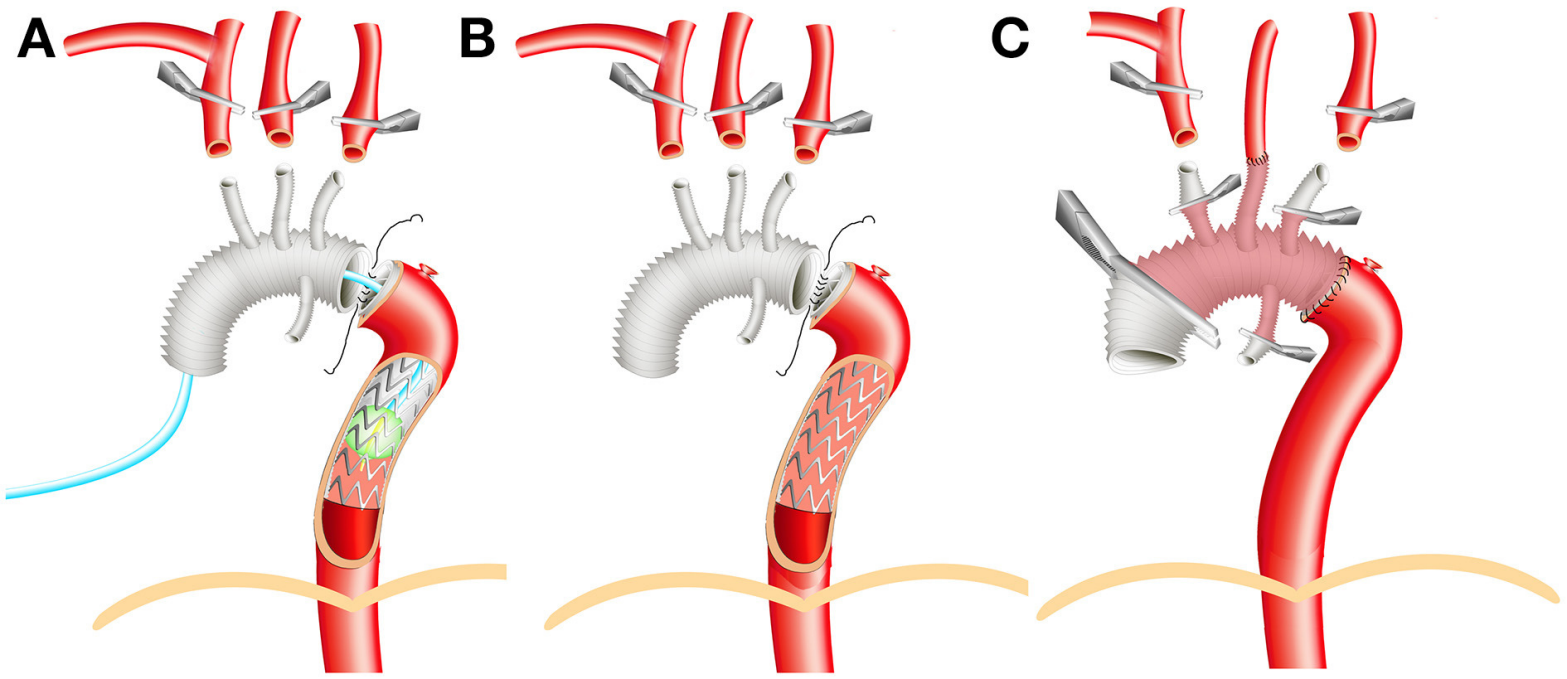
Illustration of surgical technique. **(A)** Aortic balloon occlusion. **(B)** Conventional total aortic arch replacement (TAR) with frozen elephant trunk (FET). **(C)** Aortic reconstruction before rewarming.

The sheathed aortic balloon was deployed into the stent-graft, acknowledging that any part the balloon could be constricted by the solid metal stent. The balloon was inflated via injection of 40 ml saline to compress the stent-graft. Once the balloon was fixed, lower body perfusion was restarted through the right femoral artery cannula along with ASCP through the right axillary artery cannula. The CPB flow restarted and gradually increased to 50% of the total flow. The lower body CA was 6.3 ± 5.7 min (Figure 1A). During conventional TAR with FET surgery, lower body CA is required until distal anastomosis (suture of the wall of the DTA, the proximal wall of the stent-graft, and the distal wall of trifurcated graft) is completed, before CPB can be restarted and gradually increased to 50% of the total flow (Figure 1B).

For DeBakey type III patients, with an ascending aortic aneurysm and/or intracardiac diseases (such as aortic valve regurgitation or atrial septal defect), it is possible to treat all diseases during a one-stage operation by performing TAR with FET surgery. The treatment of AD in the DTA and/or left subclavian artery is more effective in the long term compared with thoracic endovascular aortic repair. The chronicity of AD and the aortic pathology of the patients were recorded in the patient records. After matching, both the aortic pathology and chronicity classifications of AD indicated that the conventional group was more, or at least as likely to be, reasonable as the conventional group before matching. The left common carotid artery was anastomosed first, and then perfusion through both carotid arteries could be realized. The rewarming process was subsequently started (Figure 1C).

### Data Collection and Statistical Analysis

Major complications and clinical endpoints were recorded according to the consensus statement from the IAASSG (5). Specifically, postoperative acute kidney injury (AKI) was defined as a serum creatinine (Scr) volume over 1.5 times that of the normal value (<133 μmol/L). Liver injury was defined as transaminase concentrations 1.5 times greater than those of the corresponding upper range of their normal values [alanine transaminase (ALT) <40 U/L, aspartate transaminase (AST) <55 U/L] for more than 48 h. The clinical examinations were all collected preoperatively. In addition, continuous renal replacement therapy (CRRT) is indicated when the patient has a urine volume <0.5 ml/(h-kg) for more than 6 h or a pH <7.2 and residual base < -8 mmol/L or serum potassium >6.0 mmol/L or a 2- to 2.9-fold increase in serum creatinine value from baseline levels.

Following the surgical procedure, data regarding patient transfer to the intensive care unit (ICU) was collected. (0), twice on postoperative days 1 to 3 (1, 1.5, 2, 2.5, 3), mid-time point of ICU if the patient stayed in ICU more than 4 days (Mid), the last examination before transferred out from ICU (ICU), the first examination after transfer out from ICU to ward (Ward), and the last examination before discharge (Discharge). These items were considered as both time-matched comparisons during the first three postoperative days and event-matched comparisons obliterating time (preoperative, transfer out of ICU, and fully recovered discharge values) to achieve maximum power. Categorical variables were compared using the χ^2^ test. Continuous variables were presented as mean ± SD; ALT and AST were presented as mean ± SEM. All parameters related to time also included the median (M) and interquartile range. A normality test between the two groups was performed on the continuous variables. If the variable satisfied the normality test (Shapiro–Wilk test), the statistical analysis was evaluated with a *t*-test. If the variable failed the Shapiro–Wilk test, it was evaluated by the Mann–Whitney *U*-test. Clinical data were depicted in the figures to show the characteristics of the trends of perioperative alteration between the two groups. Due to fluctuating values, no statistical analyses could be performed to compare differences between time points and multiple comparison corrections to obtain *p*-values. According to the basic characteristics of comparison groups, once there are significant differences of potential confounding variables found between groups, propensity score matching will be conducted to control the confounding factors and reduce the risk of potential biases. The propensity score will be generated through a multivariable logistic regression analysis model and be applied to create 1:1 matching pairs of the conservative group and the innovative group. A nearest neighbor matching algorithm without a caliper method will be used. All the statistical analysis in the study will be performed using SPSS software (version 22.0; IBM-SPSS Inc., Armonk, NY, USA).

## Results

### Baseline Characteristics and Early Outcomes

The baseline demographic and clinical characteristics of the patients are listed in Table 1. Several differences are evident: the aortic balloon occlusion (ABO) group is associated with older age, less cardiac surgery history, and inconsistently distributed CPB time, all of which were included in the propensity score matching. The matched cohorts consisted of mostly male patients, aged 49.9 ± 12.2 and 49.6 ± 10.0 years, respectively (*p* = 0.547). The most common aortic pathology type present in both cohorts was the AD in the acute stage (<72 h). All patients had similar smoking, cardiovascular disease, and cerebrovascular event histories. There were no statistically significant differences between patients having aortic arch lesions with a proximal extension to the aortic root (43.1 vs. 42.3%, *p* = 0.827) or distal extension to iliac arteries (59.4 vs. 63.4%, *p* = 0.513) in the ABO group and the conventional group, respectively. In addition to TAR with FET operations, the concomitant root operations and coronary artery bypass grafts were performed according to the related indications in a similar proportion in both groups. Duration of lower body CA and ASCP time was significantly lower in the ABO group compared with the conventional group (1 ± 4.2 min and 6.3 ± 5.7 min, *p* < 0.001, respectively), whereas the nasal temperature was greater (25.5 ± 1.0°C and 27.4 ± 1.1°C, respectively) during CA.

**Table 1:** Preoperative and operative details.

**Variables**	**Aortic balloon occlusion (*N* = 130)**	**Conventional before matching (*N* = 230)**	***P***	**Conventional after matching (*N* = 130)**	***P***
Age	49.84 ± 12.21	48.00 ± 10.27	0.087	49.63 ± 9.97	0.547
Male	99 (76.15)	173 (75.22)	0.843	103 (79.23)	0.551
Smoke history	74 (56.92)	130 (56.52)	0.941	76 (58.46)	0.802
Mild	5 (3.85)	6 (2.61)	0.806	6 (4.62)	0.935
Heavy	69 (53.08)	124 (53.91)		70 (53.85)	
Chronic obstructive pulmonary disease	6 (4.62)	2 (0.87)	0.021	2 (1.54)	0.151
Cardiac surgery history	2 (1.54)	15 (6.52)	0.032	3 (2.31)	0.652
Coronary artery disease history	13 (10.00)	24 (10.43)	0.896	14 (10.77)	0.839
Myocardial infarction history^1^	1 (0.77)	6 (2.61)	0.225	4 (3.08)	0.176
Cerebrovascular accident^2^	11 (8.46)	23 (10.00)	0.632	12 (9.23)	0.827
Marfan syndrome	6 (4.62)	22 (9.57)	0.092	7 (5.38)	0.776
Time of onset (d)	86.04 ± 401.33 2.83 (1.23–11.90)	51.26 ± 259.972.52 (1.07–9.06)	0.373	53.27 ± 261.732.72 (1.01–10.62)	0.336
**Classification based on chronicity** ***N*** **(%)**					
Acute, <7 d (h)	50.47 ± 37.92 35.25 (23.48–66.88) 87 (66.92)	51.96 ± 42.4138.32 (22.48–69.56)168 (73.04)	0.228 (0.220)	48.62 ± 37.1131.83 (19.62–69.70)93 (71.54)	0.432 (0.420)
Subacute, 7–30 d (d)	15.10 ± 5.54 14.07 (10.40–19.11) 23 (17.69)	14.28 ± 5.7112.28 (9.4–17.58)20 (8.70)	0.638 (0.011)	14.06 ± 5.9412.23 (10.01–16.56)13 (10.00)	0.017 (0.073)
Chronic, >30 d (d)	556.21 ± 933.51 160 (54.5–372.5) 20 (15.38)	265.21 ± 565.7197 (40–213)42 (18.26)	0.161 (0.488)	271.46 ± 565.7984.5 (35.75–223.25)24 (18.46)	0.013 (0.508)
**Aortic pathology**					
Aneurysm	7 (5.38)	9 (3.91)	0.515	7 (5.83)	1
Aortic dissection	123 (94.62)	221 (96.09)		123 (94.62)	
Aortic dissection classification^3^	*N* (N/123)	*N* (N/221)		*N* (N/123)	
Stanford type A (DeBakey I&II)	105 (85.37)	208 (94.12)	0.007	119 (91.54)	0.002
Stanford type B (DeBakey III)	7 (5.69)	4 (1.81)	0.050	0 (0)	0.007
Stanford type NANB	11 (8.94)	9 (4.04)	0.064	4 (3.25)	0.062
Aortic dissection involvement	*N* (N/123)	*N* (N/221)		*N* (N/123)	
Root	53 (43.09)	93 (42.08)	0.856	55 (42.31)	0.797
Ascending	105 (85.37)	208 (94.12)	0.007	119 (91.54)	0.002
Arch	123 (100)	221 (100)	1	123 (100)	1
Total/proximal	116 (94.31)	217 (98.19)	0.050	123 (100)	0.007
Left subclavian artery or distal	7 (5.69)	4 (1.81)		0 (0)	
Thoracic descending	115 (93.50)	207 (93.67)	0.951	116 (94.31)	0.790
Total abdominal	84 (68.29)	143 (64.17)		84 (68.29)	1
Iliac	73 (59.35)	135 (61.09)	0.752	78 (63.41)	0.513
**Aortic diameter on CT**					
Ascending aorta	45.81 ± 9.96	47.53 ± 12.21	0.274	45.85 ± 11.60	0.736
Descending aorta	31.26 ± 6.38	34.03 ± 9.44	0.100	32.84 ± 7.06	0.492
Branch aortic dissection involvement	*N* (N/123)	*N* (N/221)		*N* (N/123)	
Coronary (total)	26 (21.14)	56 (25.34)	0.381	32 (26.02)	0.367
Left coronary artery	11 (8.94)	20 (9.05)	0.974	11 (8.94)	1
Left coronary artery only	1 (0.77)	4 (1.74)	0.450	3 (2.31)	0.314
Right coronary artery	25 (20.33)	52 (23.53)	0.494	29 (23.58)	0.538
Right coronary artery only	15 (11.54)	36 (15.65)	0.282	21 (16.15)	0.281
Coronary (Both)	10 (7.69)	16 (6.96)	0.796	8 (6.15)	0.625
Left renal artery involvement	60 (48.78)	110 (49.77)	0.860	67 (54.47)	0.372
Left renal artery evidence of stenosis	22 (17.89)	44 (19.91)	0.648	24 (19.51)	0.744
Right renal artery involvement	38 (30.89)	71 (32.13)	0.814	41 (33.33)	0.682
Right renal artery evidence of stenosis	17 (13.82)	27 (12.22)	0.669	14 (11.38)	0.564
Aortic root operation					
None	41 (31.54)	96 (41.74)	0.158	53 (40.77)	0.388
Repair or plasty	53 (40.77)	68 (29.57)		44 (33.85)	
Bentall	27 (20.77)	52 (22.61)		25 (19.23)	
Wheat	8 (6.15)	10 (4.35)		5 (3.85)	
David	1 (0.77)	4 (1.74)		3 (2.31)	
Concomitant coronary artery bypass graft (CABG)	18 (13.85)	30 (13.04)	0.830	16 (12.31)	0.713
Planned CABG	18 (13.85)	25 (10.87)	<0.001	14 (10.77)	<0.001
Salvage CABG	0 (0)	5 (2.17)		2 (1.54)	
Operation time	396.95 ± 85.42 379 (337–430)	409.76 ± 118.77 395 (330–463)	0.445	402.64 ± 113.06 394 (321–458)	0.872
Cardiopulmonary bypass time	185.20 ± 56.99 175 (148–201)	188.57 ± 89.25 162 (141–209)	0.152	188.12 ± 74.35 159 (141–211)	0.245
Clamp time	119.34 ± 36.25 114 (94–143)	113.05 ± 39.53 109 (89–131)	0.034	112.95 ± 38.75 107.5 (85–132.25)	0.073
Cooling time	20.90 ± 7.26 20 (16–24)	30.33 ± 9.78 29 (23–36)	<0.001	30.52 ± 10.03 29 (23–36)	<0.001
Circulatory arrest duration	6.33 ± 5.74 5 (3–7)	17.24 ± 4.36 17 (14–20)	<0.001	17.09 ± 4.18 17 (14.75–19)	<0.001
Antegrade selective cerebral perfusion time	39.88 ± 14.51 37 (31–47)	33.28 ± 10.01 32 (26–38)	<0.001	33.38 ± 10.92 32 (26–39)	<0.001
Rewarming time	53.64 ± 17.51 53 (45–62)	65.58 ± 19.52 63 (53–74)	<0.001	65.98 ± 19.60 64 (54–74.5)	<0.001
Post cardiopulmonary bypass time	128.78 ± 54.29 119 (98–137)	166.10 ± 66.00 157 (123–199)	<0.001	167.55 ± 73.08 156 (126–199)	<0.001
**The temperature when the circulatory arrest was commenced (°C)**					
Nasopharyngeal	27.41 ± 1.06	25.37 ± 1.34	<0.001	25.45 ± 0.96	<0.001
Rectal	29.00 ± 1.91	28.46 ± 2.55	0.015	28.61 ± 2.53	<0.001

*Continuous variables are expressed as mean ± standard deviation and compared by Mann-Whitney U-test except for age, which is normally distributed and compared by Student's t-test. Categorical variables are expressed as number (%) and compared using the χ^2^ test*.

1 *Myocardial infarction history is included in patients with coronary artery disease history. This is the old history and is not associated with the onset of aortic dissection, which may involve coronary arteries and may cause new myocardial ischemia/infarction*.

2 *Cerebrovascular accident includes stroke history and transient ischemic attack history*.

3 *Stanford type A (DeBakey I) is defined as ascending aortic arch involvement and distal beyond, Stanford type B (DeBakey II) as involving from the left subclavian artery or distal beyond, and the rest as none-type A-none-type B (NANB) Stanford type, which is dissection involved in the proximal aortic arch, but not ascending aorta. There were no DeBakey III patients*.

After propensity score matching, the 30-day mortality of the ABO group and the conventional group was 4.6 and 10.8% (*p* = 0.063), respectively. Although the 30-day mortality of the ABO group was lower in the ABO group, the difference was not statistically significant. The ICU stay time was similar in both groups, 119.9 ± 106.3 h vs. 131.1 ± 150.0 h (*p* = 0.415), as was the postoperative in-hospital stay time, 11.8 ± 5.4 days vs. 12.3 ± 6.7 days (*p* = 0.900). The incidence of stroke (3.1 vs. 4.6%, *p* = 0.519), temporal paraplegia (2.3 vs. 5.4%, *p* = 0.197), and delirium (3.1 vs. 7.7%, *p* = 0.099) were also similar in the ABO group compared with the conventional group, respectively.

### Renal System Results

Volumes of Scr and urine, and CRRT were found to be the most common and therefore reliable parameters to evaluate the postoperative AKI. Patients with oliguria or anuria who underwent CRRT had the worse prognosis. Scr was a key indicator when evaluating the underlying AKI incidence. The ABO technique significantly reduced the incidence of postoperative AKI (23.1 vs. 38.5%, *p* = 0.007) and CRRT (7.7 vs. 16.2%, *p* = 0.037) in the ABO group compared with the conventional group, respectively.

**Figure 2:**
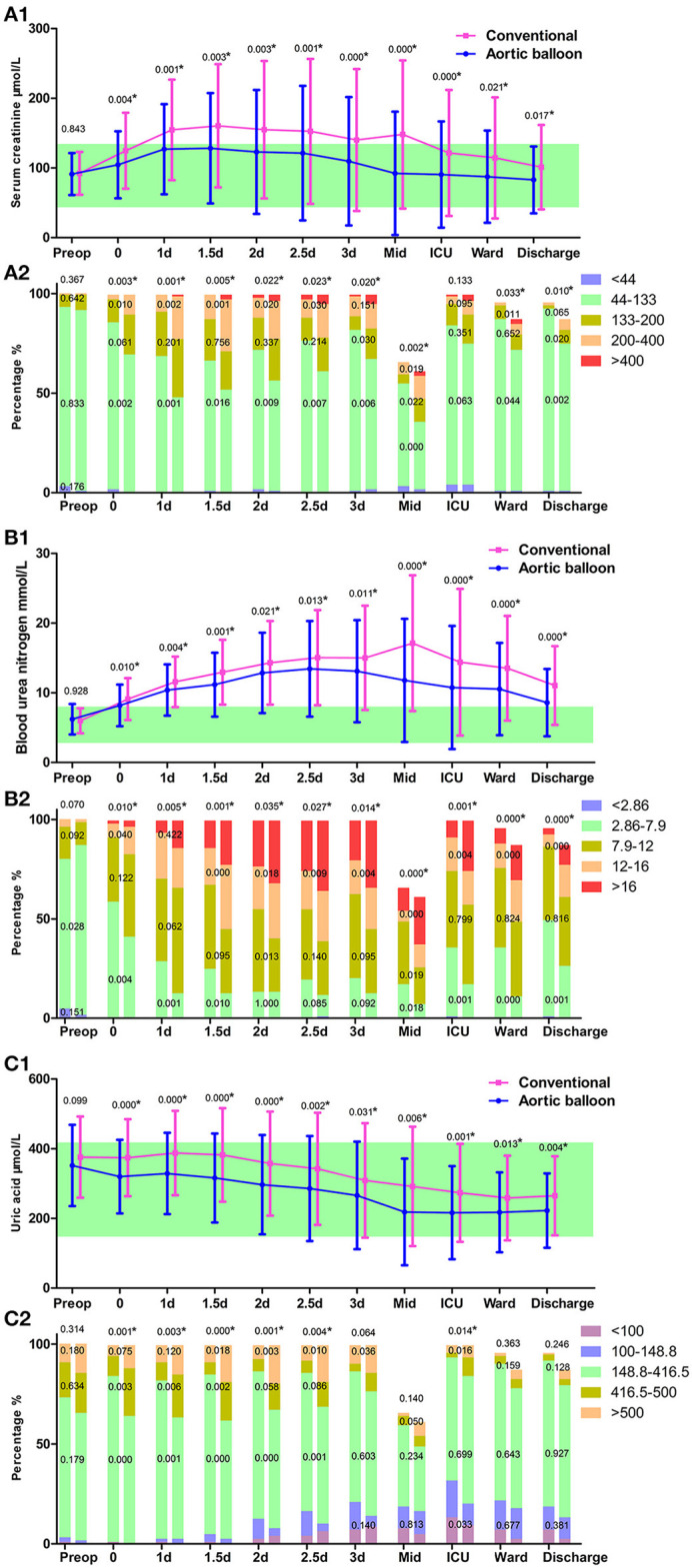
Renal function. **(A1)** The trajectory of serum creatinine (Scr). **(A2)** Scr grading in the column. **(B1)** The trajectory of blood urea nitrogen (BUN). **(B2)** BUN grading in the column. **(C1)** The trajectory of uric acid (UA). **(C2)** UA grading in the column. Data points are represented by mean with standard deviation bars. The green band represents the referred normal range of the examination. The *P*-value of the continuous variable is represented above the standard deviation bar, which is calculated by Student's t-test (normally distributed) or Mann-Whitney U test (non-normally distributed) with no multiple comparison correction to the *P*-value. The *P*-value of the categorical variable is represented in the graded column to represent for each grade, or above the grade to represent a total, which is calculated by χ^2^ test. ^*^P < 0.05.

To further investigate the in-hospital recovery course of renal function and explore the benefits of ABO technique, we examined the patients' blood examination results. Scr value reflects whether postoperative renal function recovery is good or poor. Blood urea nitrogen values were similar in pattern to those of Scr; however, the blood urea nitrogen peak appeared later. Uric acid (UA) was also recorded from the renal function examination, and it tended to decrease. The decrease in postoperative UA levels may also reflect renal function recovery (Figure 2).

### Postoperative Bleeding

The operation time after CPB was significantly shorter in the ABO group than the conventional group (128.8 ± 54.3 min vs. 167.6 ± 73.1 min, *p* < 0.001). The propensity-matched comparisons showed that the ABO group had a lower operative time and lower volumes of red blood cell (RBC) and platelet transfusions.

**Figure 3:**
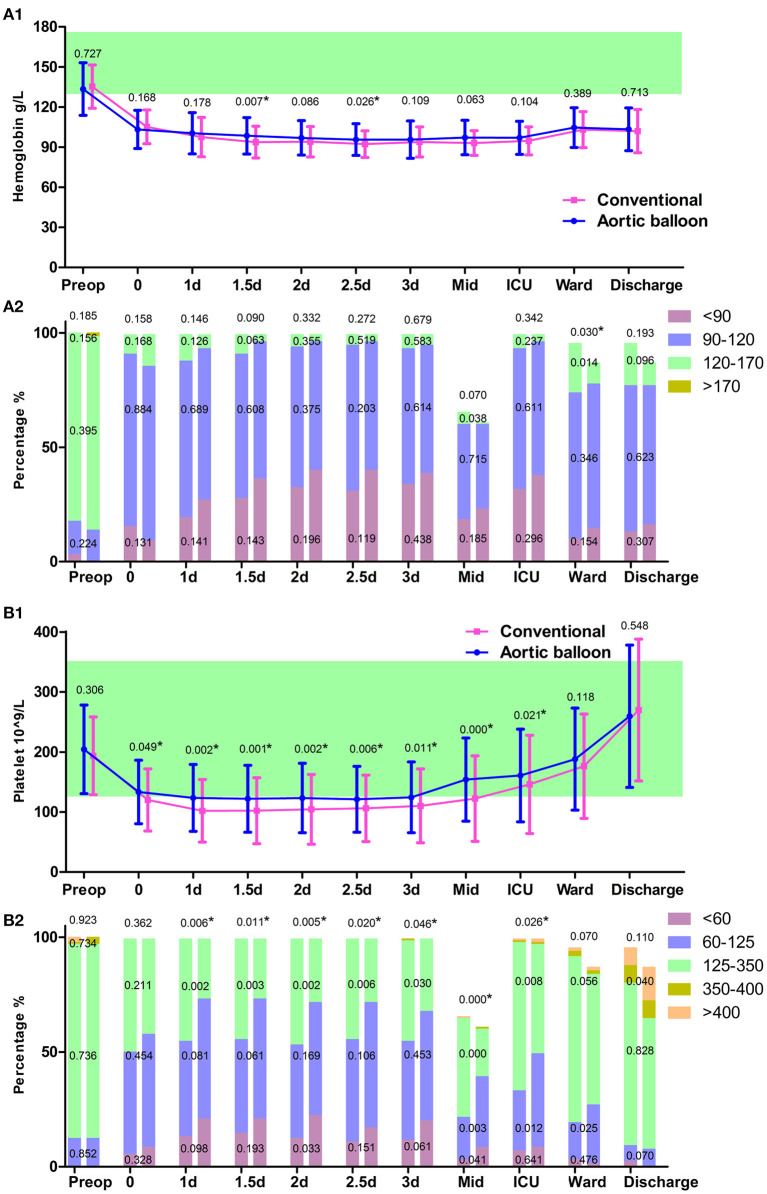
Blood routine test. **(A1)** The trajectory of hemoglobin. **(A1)** hemoglobin grading in the column. **(B1)** The trajectory of platelet count. **(B2)** Platelet count grading in the column. Data points are represented by mean with standard deviation bars. ^*^P < 0.05.

RBC transfusion is necessary when a patient's hemoglobin concentration is below 80 g/L following cardiac surgery (6). More than 50% of patients received at least one 1 unit transfusion of RBC during their in-hospital stay (Figure 3A). Plasma and platelet transfusions were used mostly intraoperative to avoid bleeding and decrease the thoracic drainage output. Platelet level dropped drastically after the operation, especially in the conventional group, which may be caused by blood loss and/or heparin-induced thrombocytopenia (Table 2). In addition, compared with the ABO group, more patients in the conventional group required a platelet transfusion (13.9 vs. 26.9%, respectively, *p* = 0.009). Platelet levels showed an obvious increase after postoperative day 3, and no further platelet transfusions were necessary (Figure 3B).

**Table 2:** The early outcomes.

**Variables**	**Aortic balloon occlusion (*N* = 130)**	**Conventional before matching (*N* = 230)**	***P***	**Conventional after matching (*N* = 130)**	***P***
30-day mortality	6 (4.62)	18 (7.83)	0.241	15 (10.77)	0.063
Wake-up time (h)	15.16 ± 23.56 8.12 (4.92–13.80)	20.07 ± 26.49 11.75 (7.80–19.02)	<0.001	23.52 ± 32.71 11.92 (7.85–22.32)	<0.001
Mechanical ventilation (h)	34.13 ± 40.97 18.67 (13.30–36.87)	46.74 ± 106.69 21.48 (13.47–46.97)	0.217	48.93 ± 87.83 21.70 (13.52–55.23)	0.133
Prolonged mechanical ventilation (>72 h)	11 (8.46)	42 (18.26)	0.012	28 (21.54)	0.003
ICU stay (h)	119.90 ± 106.32 90.53 (59.33–132.75)	119.69 ± 123.36 86.38 (58.67–134.15)	0.380	131.14 ± 150.00 86.38 (44.32–136.58)	0.415
Postoperative in-hospital stay (d)	11.80 ± 5.35 11 (8–14)	12.42 ± 6.03 11 (9–14)	0.321	12.34 ± 6.70 10 (8–14)	0.900
Acute kidney injury	30 (23.08)	82 (35.65)	0.013	50 (38.46)	0.007
Continuous renal replacement therapy	10 (7.69)	23 (10.00)	0.466	21 (16.15)	0.037
Stroke	4 (3.08)	9 (3.91)	0.683	6 (4.62)	0.519
Temporal paraplegia	3 (2.31)	10 (4.35)	0.319	7 (5.38)	0.197
Delirium	4 (3.08)	15 (6.25)	0.160	10 (7.69)	0.099
Hepatic injury	16 (12.31)	64 (27.83)	0.001	39 (30.00)	<0.001
Acute pancreatitis	6 (4.62)	22 (9.57)	0.091	13 (10.00)	0.095
Severe lung infection	3 (2.31)	18 (7.83)	0.032	11 (8.46)	0.028
Re-exploration for post-operative bleeding	7 (5.38)	9 (3.91)	0.515	8 (6.15)	0.790
Left recurrent nerve injury	4 (3.08)	1 (0.008)	0.040	1 (0.004)	0.176
**Total chest tube drainage of 0–3 postoperative day (ml)**					
Operation day	429.15 ± 585.05	403.21 ± 264.47	0.760	432.72 ± 309.23	0.484
Postoperative day 1	321.47 ± 201.07	381.23 ± 213.73	0.001	375.35 ± 218.95	0.006
Postoperative day 2	211.34 ± 192.45	269.51 ± 179.42	<0.001	259.13 ± 175.19	0.001
Postoperative day 3	176.03 ± 143.73	223.86 ± 148.50	<0.001	213.29 ± 130.12	0.003
Prothrombine X (IU) usage	1144.26 ± 293.47 122 (93.85)	1303.51 ± 401.08 228 (99.13)	<0.001 (0.003)	1321.19 ± 381.82 119 (91.54)	<0.001 (0.475)
Novoseven (mg) usage	2.11 ± 0.56 83 (63.85)	2.23 ± 0.64 147 (63.91)	0.084 (0.990)	2.12 ± 0.66 78 (60.00)	0.863 (0.523)
**Transfusion requirement (average per patients and percentage of usage)**					
Red blood cell (u) total	4.98 ± 6.53 83 (63.85)	6.47 ± 8.51 174 (75.65)	0.042 (0.017)	7.28 ± 10.41 99 (76.15)	0.008 (0.030)
Operative	2.06 ± 3.15 55 (42.31)	3.02 ± 4.53 117 (50.87)	0.090 (0.118)	3.64 ± 5.19 74 (56.92)	0.009 (0.018)
Postoperative	2.92 ± 4.50 65 (50.00)	3.45 ± 6.18 132 (57.39)	0.351 (0.176)	4.17 ± 7.55 80 (61.54)	0.101 (0.061)
Plasma (ml) total	488.37 ± 573.32 82 (63.08)	614.78 ± 833.55 141 (61.30)	0.397 (0.739)	725.58 ± 992.81 86 (66.15)	0.120 (0.604)
Operative	302.33 ± 399.31 71 (54.62)	367.83 ± 432.13 124 (53.91)	0.292 (0.898)	400.00 ± 445.11 77 (59.23)	0.089 (0.452)
Postoperative	186.05 ± 354.60 42 (32.31)	246.96 ± 576.29 73 (31.74)	0.887 (0.912)	325.58 ± 716.36 47 (36.15)	0.337 (0.513)
Platelets (u) total	1.16 ± 0.99 102 (78.46)	1.40 ± 1.53 189 (82.17)	0.245 (0.390)	1.62 ± 1.84 112 (86.15)	0.049 (0.104)
Operative	0.86 ± 0.66 93 (71.54)	0.92 ± 0.90 172 (74.78)	0.675 (0.502)	0.95 ± 0.99 99 (76.15)	0.595 (0.397)
Postoperative	0.29 ± 0.84 18 (13.85)	0.48 ± 1.28 51 (22.17)	0.078 (0.054)	0.68 ± 1.62 35 (26.92)	0.015 (0.009)

*Continuous variables are expressed as mean ± standard deviation and compared by the Mann-Whitney U-test. Categorical variables are expressed as number (%) and compared using the χ^2^ test*.

### Respiratory System and Inflammation

**Figure 4:**
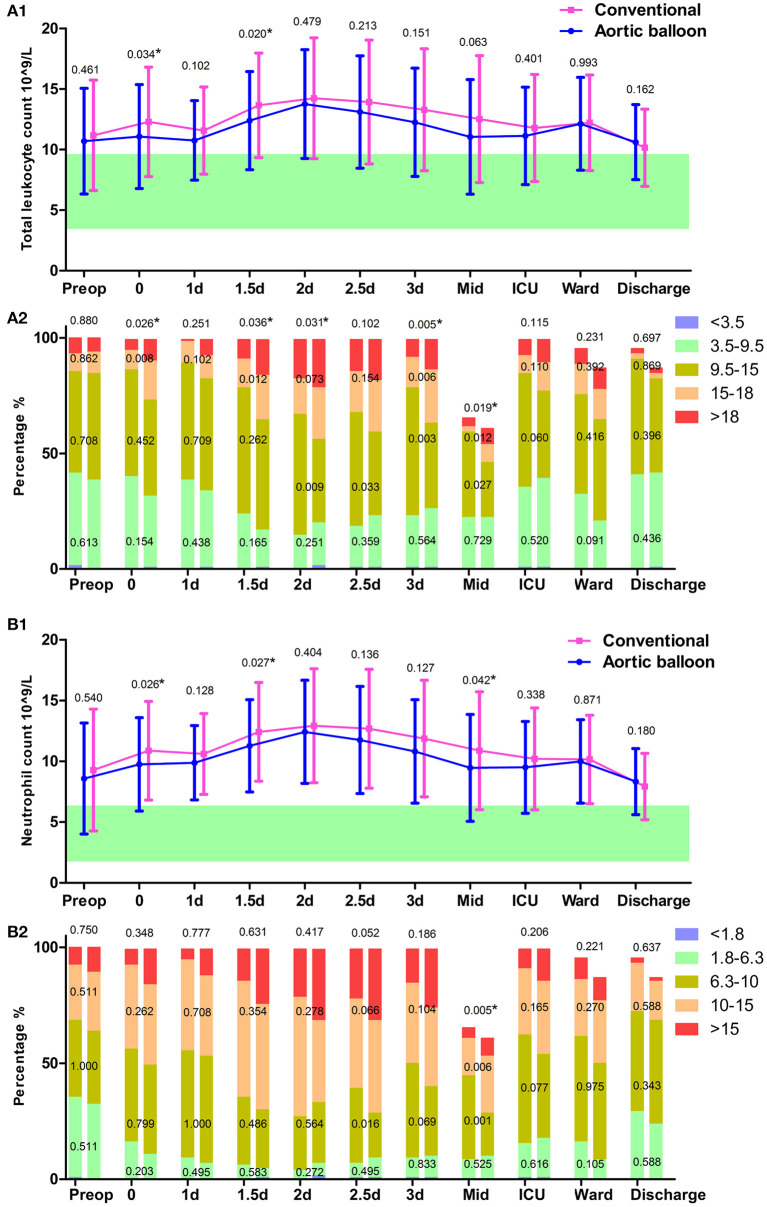
Leukocyte test. **(A1)** The trajectory of total leukocyte count. **(A2)** Total leukocyte count grading in the column. **(B1)** The trajectory of neutrophil count. **(B2)** Neutrophil count grading in the column. Data points are represented by mean with standard deviation bars. ^*^P < 0.05.

After transfer to the ICU, patients frequently regained consciousness before the mechanical ventilation was withdrawn. The incidence of lung infection was lower in the ABO group compared with the conventional group (2.3 vs. 8.5%, respectively, *p* = 0.028). The postoperative wake-up time was much shorter in the ABO group compared with the conventional group, at 15.2 ± 23.6 h vs. 20.1 ± 26.5 h, respectively (*p* < 0.001), which may be because the superior central nervous system was well-protected via the ABO technique to a certain extent. The mechanical ventilation support time was 34.1 ± 41.0 h and 48.9 ± 87.8 h (*p* = 0.133). The ABO group had short mechanical ventilation support time, 8.5% vs. 21.5% (*p* = 0.003) in the ABO group compared with the conventional group, respectively. The leukocyte counts revealed both neutrophil and total leukocyte counts were higher in the conventional group during the initial postoperative period (Figure 4). The neutrophil and total leukocyte counts dropped to preoperative levels before discharge, suggesting a favorable recovery during the in-hospital stay.

### Hepatic System Results

**Figure 5:**
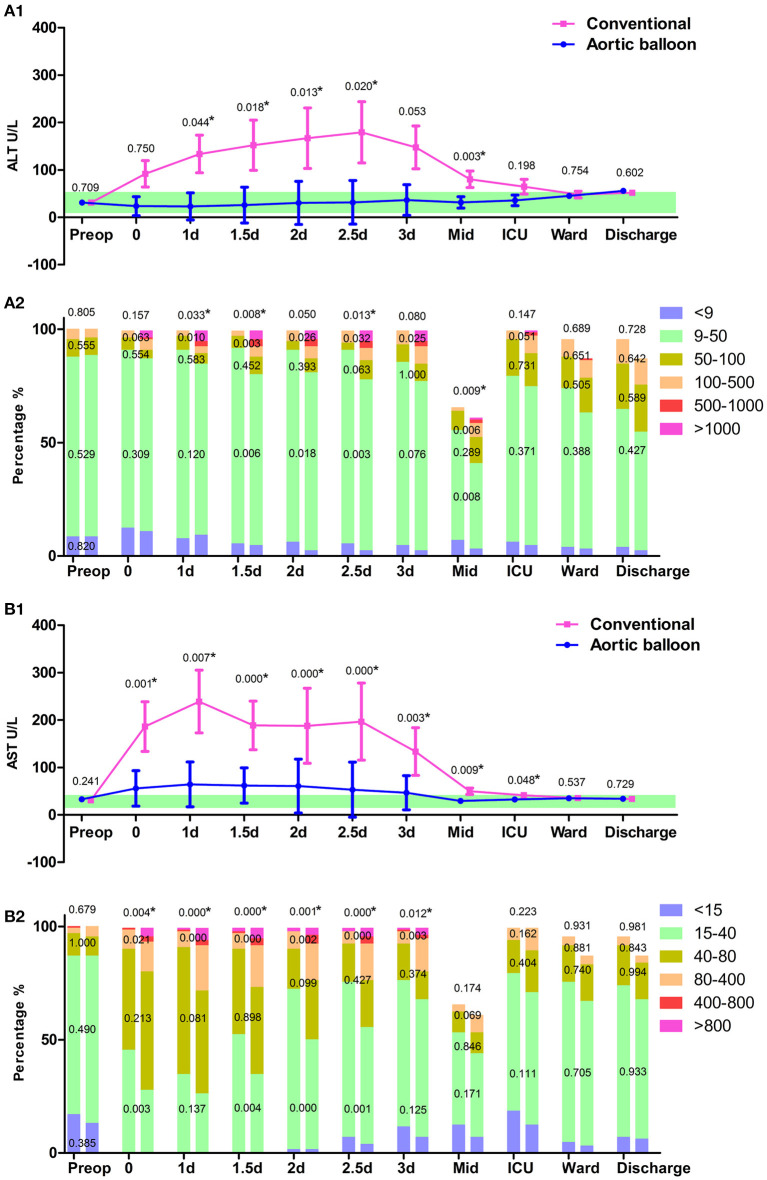
Hepatic function. **(A1)** The trajectory of alanine transaminase (ALT). **(A2)** ALT grading in the column. **(B1)** The trajectory of aspartate transaminase (AST). **(B2)** AST grading in the column. Data points are represented by mean with a standard error of mean bars. ^*^P < 0.05.

Hepatic complications were reflected by postoperative transaminase, and the ABO group had a lower incidence of hepatic injury (12.3 vs. 30.0%, *p* < 0.001) than the conventional group. The average level of ALT progressively increased in the ABO group during the in-hospital stay, and the conventional group rapidly increased in the first three postoperative days and fell to a similar level to that of the ABO group. Both groups had a sharp growth in average levels of AST during the ICU stay, and these gradually recovered to normal levels after patients were transferred back to the ward (Figure 5). The tendency of ALT and AST, therefore, played an important role in indicating hepatic injury.

## Discussion

In our institution, TAR with FET is the standard method to treat AD and other complexed aortic arch diseases (2); it is a safe and effective strategy (7) with promising long-term outcomes (3). Recently, the ABO technique was applied to TAR with FET. Its use consistently shortened the lower body CA time and raised the target nadir temperature, improving organ protection during the operation. Due to the complexity of postoperative complications, multi-system real-time surveillance is required during the ICU stay (8). This study investigated the effects of the ABO technique on the clinical outcomes, through continuous monitoring of patients' blood examination results during the in-hospital stay.

Renal injury was defined by Scr increase or urine output decrease. In our results, all oliguria and anuria received CRRT treatment, and AKI was graded by Scr level. Scr levels in both groups rose during the stay in the ICU but had recovered to preoperative levels before discharge. The postoperative Scr levels in the ABO group were consistently lower than that in the conventional group. The ABO technique may improve blood perfusion in the kidneys. Increasing the CA temperature alone cannot provide renal protective effects if CA time is not shortened (9).

Over the past few decades, transfusions in our hospital have greatly improved. Significant improvements have been achieved using the ABO technique, with a reduction in RBC, plasma, and platelet transfusion volumes, as well as reduced bleeding control times and thoracic drainage output. This benefit may be due to an improved postoperative coagulation function strategy, which may also be related to the use of the ABO technique. A previous study found that a decrease in patients' postoperative platelet counts was associated with the severity of AKI (10). A rebound in the number of platelets was also observed in the current study, as indicated by the persistent platelet activation and aggregation in the initial postoperative period (10). The postoperative platelet count may increase to such a high level that anti-platelet therapy could be actively proposed following a suitable evaluation. Moreover, the FET procedure was considered as a risk factor that may increase the incidence of spinal cord ischemia (11). Thus, anti-platelet therapy and dynamic assessment of limb movement should be considered in patients with high postoperative platelet counts to reduce the risk of thrombi.

The hepatic function was also significantly improved by the ABO technique; the AST was significantly higher in the conventional group and the ALT increased steadily during the in-hospital stay and exceeded the AST level before discharge. Previous studies highlight how AST is more sensitive than ALT in predicting postoperative hepatic injury (12–14). In our investigation, the AST level was high during the initial postoperative period, although it returned to normal levels following transfer out of the ICU. This demonstrates that AST was a more sensitive indicator to evaluate the hepatic injury suffered from the CPB procedure, while ALT was more strongly associated with drug-induced hepatic injury. Although ALT levels increased during the in-hospital stay in line with drug usage, most cases did not need specific hepatoprotective treatment (15).

The ABO technique significantly shortened the postoperative wake-up time, which may provide an improved protective effect on the central nervous system, although this hypothesis needs further investigation. In addition, the subsequent mechanical ventilation support time was shortened, and the pulmonary infection rate was also low.

This study has several limitations. First, this is a retrospective study and we only presented the data within a short period, mainly because the technique was only invented in August 2017, although this makes the standard of ICU care included in the study as uniform as possible. Second, as a historical comparison, the influence of increasing team experience may also create potential bias. Third, propensity score matching is well-performed but could not adjust differences for every indicator analyzed in this study, and results of some comparisons may have been impacted. In addition, as for this new technique we introduced, it is not suitable for the following situations: where both femoral arteries are not suitable for cannulation; the DTA has formed a large aneurysm at the suture level of the trifurcated graft; or where Cronus stents have been implanted. There is still space for improvement of the new technique and we will continue our study in the future.

## Conclusions

We systematically introduced a new ABO technique, based on the primary clinical outcomes, and this new method is not inferior to the traditional approach. Furthermore, some improved clinical outcomes were seen in the ABO group. Although there were no significant statistical differences between the two groups, in some respects, the clinical outcomes of the ABO group were improved compared with those of the conventional group. However, the sample size of the current study is small. We hope to enroll more patients to further investigate the value in the clinical application of this new technique.

The ABO technique may serve as an important operative method that improves the blood perfusion of organs and the recovery process during the in-hospital stay. However, the aortic balloon occlusion technique cannot be seen as a perfect technique that avoids all complications. We believe that continuous perfusion via the ABO technique is crucial in diminishing operative injury rates in high-risk patients. Therefore, using this new technique in TAR–FET may encourage cardiac surgeons to aim for improved clinical outcomes in this complex group of patients.

## Data Availability Statement

The raw data supporting the conclusions of this article will be made available by the authors, without undue reservation.

## Ethics Statement

The studies involving human participants were reviewed and approved by the Institutional Ethics Committee of Fuwai Hospital, National Center for Cardiovascular Diseases, Chinese Academy of Medical Sciences, and Peking Union Medical College (No: 2018-1069). Written informed consent for participation was not required for this study in accordance with the national legislation and the institutional requirements.

## Author Contributions

LW and XS: conception and design. ZC: administrative support. YL and SL: provision of study materials or patients. LW: collection and assembly of data. LW and JL: data analysis and interpretation. LW, ZC, YL, JL, HG, SL, and XS: article writing and final approval of article. All authors contributed to the article and approved the submitted version.

## Conflict of Interest

The authors declare that the research was conducted in the absence of any commercial or financial relationships that could be construed as a potential conflict of interest.

## Publisher's Note

All claims expressed in this article are solely those of the authors and do not necessarily represent those of their affiliated organizations, or those of the publisher, the editors and the reviewers. Any product that may be evaluated in this article, or claim that may be made by its manufacturer, is not guaranteed or endorsed by the publisher.

## Abbreviations

ABO: aortic balloon occlusion
TAR: total arch replacement
FET: frozen elephant trunk
CA: circulatory arrest
RBC: red blood cell
AD: aortic dissection
IAASSG: international aortic arch surgery study group
CPB: cardiopulmonary bypass
ASCP: anterograde selective cerebral perfusion
DTA: descending thoracic aorta
AKI: acute kidney injury
CRRT: continuous renal replacement therapy.
